# Do athletes alter their running mechanics after an Achilles tendon rupture?

**DOI:** 10.1186/s13047-017-0235-0

**Published:** 2017-11-28

**Authors:** Daniel Jandacka, Julia Freedman Silvernail, Jaroslav Uchytil, David Zahradnik, Roman Farana, Joseph Hamill

**Affiliations:** 1 Department of Human Movement Studies, Human Motion Diagnostic Center, University of Ostrava, Varenska 40 A, 70200 Ostrava, Czech Republic; 2 Department of Kinesiology and Nutrition Sciences, University of Nevada Las Vegas, Las Vegas, USA; 3 Department of Kinesiology, University of Massachusetts, Amherst, USA

**Keywords:** Achilles tendon, Injury, Ultrasonography, Knee

## Abstract

**Background:**

Over the past thirty years, there has been dramatic increase in incidence of Achilles tendon rupture in the athletic population. The purpose of this study was to compare the lower extremity mechanics of Achilles tendon ruptured runners with healthy controls.

**Methods:**

The participants with a past history of an Achilles tendon repair (*n* = 11) and healthy control (*n* = 11) subgroups were matched on sex, age, type of regular physical activity, mass, height, footfall pattern and lateral dominancy. Running kinetics and kinematics of the ankle, knee and hip were recorded using a high-speed motion capture system interfaced with a force platform. Achilles tendon length was measured using ultrasonography. Main outcome measures were lower extremity joint angles and moments during stance phase of running and Achilles tendon lengths.

**Results:**

Athletes from Achilles tendon group had an affected gastro-soleus complex. Athletes with history of Achilles tendon rupture had reduced ankle range of motion during second half of the stance phase of running (Δ7.6°), an overextended knee during initial contact (Δ5.2°) and increased affected knee range of motion (Δ4.4°) during the first half of stance phase on their affected limb compared to the healthy control group. There was a 22% increase in the maximal hip joint moment on contralateral side of the Achilles tendon group compared to the healthy controls.

**Conclusion:**

These results suggest a compensation mechanism, relatively extended knee at initial ground contact against the deficit in the muscle-tendon complex of the triceps surae. Overextension during sporting activities may place the knee at risk for further injury. Avoidance of AT lengthening and plantarflexion strength deficit after surgery and during rehabilitation might help to manage AT rupture since these factors may be responsible for altered running kinematics.

## Background

The Achilles tendon (AT) played a crucial role in the evolution of early humans enabling them to run faster [1]. The AT’s important role is to provide sufficient plantar flexor power in running activities. Over the past thirty years, there has been dramatic increase in incidence of AT rupture (from 2 to 22 per 100,000 person-years) primarily in the athletic population [2, 3]. Despite all medical efforts, athletes with history of AT rupture have been shown to have a substantially decreased performance in sports with running and jumping activities [4, 5]. Although up to 30% of these athletes end their sporting career after rupturing their AT, many manage to return to a physically active lifestyle [4, 5].

Evidence has been reported that individuals with a history of AT rupture have decreased ankle joint proprioception, decreased plantar flexor muscle volume, increased AT length and affected AT stiffness [6–8]. The changes in mechanical, anatomical and/or neuromuscular properties of the triceps surae lead to Achilles tendon weakness in a plantar flexed position [9], an increase plantar flexor muscle activity during locomotion [10] and reduced plantar flexor endurance even several years after rupture [11]. In addition, athletes with a previous AT rupture were 176 times more likely to suffer a contralateral Achilles tendon rupture compared to an individual without a previous AT rupture [12]. In this study, the authors hypothesized that the increased injury risk is a result of a genetic predisposition or degeneration and atrophy of the contralateral tendon. However, they did not consider the possibility of mechanical overload as a possible cause of injury.

There have been a number of published studies in the literature that reported biomechanical deficiencies in individual subsequent to an AT injury. Using the Achilles tendon total rupture score, it was reported that 50 % of individuals with a history of AT rupture suffered from post-injury problems (decreased strength, fatigue, stiffness or pain in the calf) and functional declines during physical activity at long-term follow-up [12, 13]. In a previous a case study, it was reported that a four-year running training program did not reduce the biomechanical consequences of the Achilles tendon rupture such as loading behavior and position of the ankle during the stance phase of running [14, 15]. In their case study of shod running, Jandacka et al. [14] suggested an association between AT elongation and increased dorsiflexion during initial contact of stance phase and reduced plantarflexion on the AT affected limb during toe off [14]. Only one study investigated the biomechanics of walking, light jogging and hopping of AT ruptured participants [16]. They found increased knee joint loads on the affected side during the jogging and hopping when comparing the affected and unaffected sides. Willy et al. [16] concluded that patients after an AT rupture may be at a greater risk of overuse injuries to the patellofemoral joint and knee extensors during light running. However, to our knowledge, there have been no studies that compared differences between AT ruptured athletes and healthy control group.

Therefore, the purpose of this study was to compare the lower extremity mechanics of AT ruptured runners with healthy controls (CTRL). Based on previous case reports and side to side comparisons [14–16] we hypothesized that, compared to the healthy control group the Achilles tendon group would have: 1) increased dorsiflexion at initial contact (IC); 2) reduced ankle angle range of motion on the AT affected limb during the second half of the stance phase; and 3) reduced maximum ankle and increased knee joint moments.

## Methods

### Participants

The experimental sample consisted of 22 individuals (16 males and 6 females) aged between 22 and 50 (see Table 1). Data on 11 participants were collected after recovery from Achilles tendon rupture (AT group) and 11 matched control (CTRL group) individuals. A post hoc power analysis was conducted on selected parameters (i.e. ankle range of motion, vertical impact peak, peak ankle moment) from the current study to determine if the sample size was suitable to detect true differences between the groups. Using a minimum power of 80% and an alpha level of 0.05, the post hoc analysis indicated that a sample of eight participants would be needed to detect true differences. Therefore, using a sample greater than necessary, the sample used in this study was sufficient to detect true differences.

**Table 1:** Average values (SD) and comparison of the matching and control characteristics for the Achilles tendon (AT) and healthy control (CTRL) groups

	AT (*n* = 11)	CTRL (*n* = 11)	*P*
Matching characteristics			
Age (years)	34.5 (8.3)	33.6 (7.7)	0.813
Mass (kg)	71.7 (11.1)	72.2 (11.6)	0.917
Height (m)	174.1 (9.6)	174.0 (8.7)	0.982
Initial Ankle Angle (°)	77.2 (10.9)	76.6 (15.2)	0.912
Fat (%)	18.9 (5.5)	16.9 (6.1)	0.427
Physical activity	5.1 (0.6)	5.1 (0.8)	0.772
Control characteristics			
ATRS	72.8 (21.8)	95.7 (6.4)	**0.003**
Silfverskiöld test (°)	35 (6)	41 (9)	**0.036**
Plantarflexion strength (Nm)	92 (27)	115 (25)	**0.021**
AT length difference (mm)	16.9 (9.2)	2.9 (2.3)	**0.000**
Circumference of the shank (mm)	34.9 (1.8)	36.3 (1.2)	**0.040**

The bold values indicate statistical significance at the *p* = 0.05 level. Initial Ankle Angle was taken from left ankle at instant of initial contact during shod running. ATRS means Achilles tendon total rupture score [13]. Silfverskiöld test indicate increased passive affected ankle dorsiflexion of the AT group [17]. Plantarflexion strength indicate maximal isometric plantarflexion strength on affected side of the AT group and matched ankle of the CTRL group [23]. AT length difference means absolute value of length difference between left and right AT. Physical activity evaluated according to 6-graded scale classification system of physical activity [18]

Participants in the AT and healthy CTRL groups were matched on age (maximum 2 years’ difference between AT affected and healthy control participant), type of regular physical activity (soccer, running, floorball etc.), mass (maximum 2 kg difference), height (maximum 0.02 m difference), footfall pattern (forefoot, midfoot or rearfoot) and limb dominancy. The footfall pattern of the participants was initially self-reported and then verified using a video analysis. The inclusion criteria for the AT group were: (1) at least two years after total AT rupture; (2) performing regular physical activity including running before as well as after recovery from AT rupture; (3) no current or past history of physical deformities, neurological disorders, diabetes mellitus or previous lower limb surgeries; and (4) treatment of Achilles tendon via mini-open suturing or formal open suture [17]. The Achilles tendon rupture occurred in all participants in the AT group during a sport activity. The mean time between injury and the first laboratory visit for the AT group was 72 ± 36 months. For the healthy control participants, the inclusion criteria were: (1) no current or past AT injury; (2) no current or past history of physical deformities, neurological disorders, diabetes mellitus or previous lower limb surgeries; and (3) free of medical care at the time of measurement.

### Experimental set-up

Running kinematics of the ankle, knee and hip were recorded using a high-speed motion capture system (Qualisys Oqus 100, AB, Göteborg, Sweden). Two force plates (Kistler 9286 AA, Kistler Instruments AG, Winterthur, Switzerland) were used to collect ground reaction force (GRF) data. The force plates were built into a 17 m long runway. Kinematics and ground reaction force data were sampled at a frequency of 240 Hz and 1200 Hz respectively. Running speed was controlled using the two photocells (EGMedical s.r.o., Brno, Czech Republic), located at intervals of 3 m along the runway.

### Protocol

Each participant visited the laboratory on two occasions. In the first session, an initial interview regarding their footfall pattern, weekly running distance and, for the AT group, participant’s description of injury, was completed. An Achilles tendon total rupture score questionnaire was used to investigate the outcome related to AT symptoms and physical activity [13, 18]. Body mass and body fat were determined using the segmental body composition analyzer (Tanita 418 MA, Arlington Heights, IL, USA). Modified Silfverskiöld test was used to measure ankle position using a goniometer while the participant was sitting with the foot hanging off the edge of the examination table and knee was fully extended [17]. In addition, the dominant limb was established as the limb used to kick a ball [19].

**Fig. 1:**
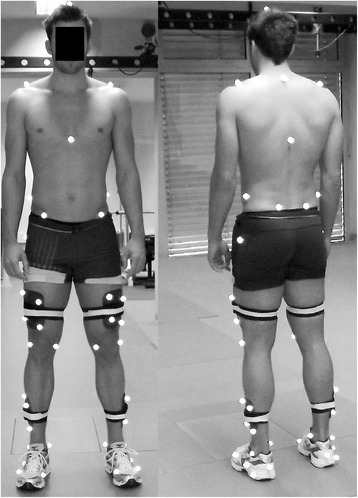
Lower extremity retro-reflective marker placement used during three-dimensional motion trials. The calibration markers were placed bilaterally on the lateral and medial malleolus, the medial and lateral femoral epicondyles, the greater trochanter of the femur, and on the feet over the first and fifth metatarsal heads. Tracking markers were positioned on the iliac spines, the anterior and posterior superior iliac crests, the acromion process, cervical vertebrae 7, thoracic vertebrae 10, Sternum Xiphisternal Joint, proximal end of head and three on the posterior aspect of the foot. Additionally, four hard light-weight plates each with four tracking markers were placed on the right and left thighs and shanks

Before data collection, each participant was fitted with 48 retro-reflective markers (see Fig. 1) [20–22]. Each participant completed a five-minute warm-up prior to data collection. Subsequently they completed five trials of shod running (Mizuno Crusader) over the force platforms at a fixed speed of 3.2 m/s (±5%). Finally, bilateral maximal isometric plantarflexion strength was measured [23].

In the second session, AT length was measured using a non-invasive method combining ultrasonography and a motion capture system [24]. The participants lay with their ankle resting in a relaxed position at the edge of the table. Two markers were positioned on the center of the ultrasound probe directly over the right and left edges of the sonogram scan area. The ultrasound image was acquired using a diagnostic ultrasound system (Mindray Bio Medical Electronics CO., LTD, Shenzhen, China) in B-mode, 10mHz, with a 75L38EA linear transducer probe. The osteotendinous and musculotendinous junctions were identified in separate images and consequently were identified in a motion analysis system as the Achilles tendon length. The distance between markers over gastrocnemius musculotendinous junction and the calcaneal osteotendinous junction were determined in Qualisys Track Manager (Qualisys, Göteborg, Sweden). The average from three measurements was used for further analysis [15].

### Data analysis

Marker trajectory and force data were processed using Visual3D software (C-motion, Rockville, MD, USA). All lower extremity segments were modelled as a frustum of right circular cones while the trunk, and pelvis were modelled as cylinders [25]. Gait events (on and off the force platform) were based on threshold vertical force value of 15 N. Kinematics and kinetic data were filtered using a fourth-order Butterworth low-pass filter with a cut-off frequency of 12 Hz and 50 Hz respectively. The local coordinate systems and the distal and proximal ends of the lower extremity segments and pelvis were derived from the standing calibration trial [26]. Subsequently, hip, knee and ankle 3-D joint angles were calculated using an Xyz Cardan rotation sequence and normalized to the standing calibration position [27]. The net hip, knee and ankle joint moments in the sagittal plane were calculated using a Newton-Euler inverse dynamics technique [26]. All joint moments were normalized to body mass.

Sagittal plane lower extremity joint angles and moments at the instant of initial contact (IC), 50% of stance phase (MID) and toe off (TO) were determined. Subsequently, the change in angles (RoM) and moments from IC to MID and MID to TO were calculated. Stance phase was divided into a weight acceptance and push-off phases based on the knowledge that, during the first half (weight acceptance) of the stance phase, the gastrocnemius transfers energy from the distal to proximal joints to help dissipate the mechanical energy of the body [28]. In addition, maximal values of net lower extremity joint moments were determined. The active peak of the vertical ground reaction force component (VGRF) and time to maximal VGRF were determined from force data. The datasets used and analysed during the current study are available from the corresponding author on reasonable request. A symmetry index (SI) for all variables was also calculated [29].

### Statistical analysis

The dependent variables, lower extremity angles at initial contact, lower extremity joint range of motion (from IC to MID and from MID to TO), the maximal joint moments, the change in lower extremity joint moments (from IC to MID and from MID to TO), maximal VGRF and time to maximal VGRF were analyzed for both lower extremities of all subjects. Using a Wilcoxon’s signed rank test, the injured lower extremity of the AT group was compared to the matched lower extremity of the healthy control group and the contralateral lower extremity of the AT group was compared to the respective lower extremity of the healthy control group. In addition, the SI for all dependent variables except of the maximal joint moments was also compared between AT and healthy control group by Wilcoxon’s signed rank test. An a priori alpha level was set as 0.05. Effect Size (ES) was calculated to determine the differences between the AT ruptured and control groups [30]. Cohen [30] proposed that ES higher than 0.5 represents a practically significant difference. All statistical analyses were performed using PASW statistics (Version 18; SPSS, Chicago, IL, USA).

## Results

The AT group reported significantly greater side-to-side differences in AT length and the Silfverskiöld test indicated an elongated gastro-soleus complex. Further analysis showed that eight participants from the AT group had an elongated Achilles tendon (Table 1). In addition, the AT group reported significantly lower plantarflexion strength and lower circumference of the shank on affected side (Table 1). There was no maximal isometric plantarflexion strength difference between the groups on unaffected side (*P* ≥ 0.05; ES ≤ 0.5). Additionally, the AT group reported lower self-reported outcome (ATRS 73/100) indicates some limitation/difficulty with various activities including running.

**Fig. 2:**
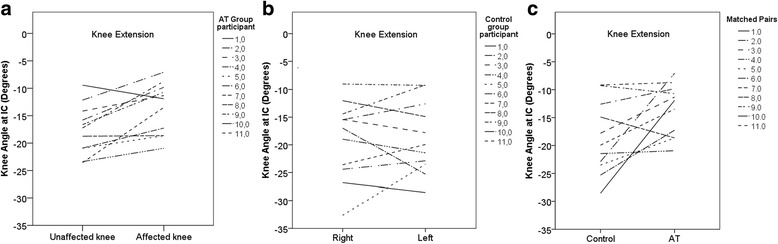
Comparison of the knee angle during the initial contact of the stance phase between the unaffected and affected limbs of the Achilles tendon group (left); the right and left limbs of the control group (middle); and the control group matched limb and the Achilles tendon affected limb (right)

During the initial contact of the stance phase, the affected lower extremity of the AT group exhibited lesser knee flexion by 5.2°, 95% CI [0.5, 10.3] compared to the matched lower extremity of the CTRL group (Fig. 2; *P* ≤ 0.05; ES ≥ 0.5). There was a statistically significant greater range of motion at the knee of the AT group on their affected limb by 4.4°, 95% CI [0.4, 8.8] during the weight acceptance phase (Table 2; *P* ≤ 0.05; ES ≥ 0.5). In addition, there was a less range of motion for the ankles of AT group (ES ≥ 0.5), however, it was significantly less (7.6 °, 95% CI [0.6, 15.7]) only on the affected limb during the push-off phase (Table 2; *P* ≤ 0.05; ES ≥ 0.5).

**Table 2:** Comparison of kinematics and kinetics during overground running between the Achilles tendon (AT) and healthy control (CTRL) groups

	Affected AT /matched control					Unaffected AT /matched control				
	AT	CTRL	∆	*P*	*ES*	AT	CTRL	∆	*P*	*ES*
Ankle										
Angle at IC (°)	77.2 (10.9)	76.6 (15.2)	0.6	0.657	0.04	77.3 (9.3)	75.7 (14.2)	1.6	0.929	0.11
Angle RoM IC to MID (°)	13.8 (11.8)	16.5 (14.1)	−2.7	0.182	0.19	13.4 (10.4)	15.6 (12.5)	−2.2	0.248	0.18
Angle RoM MID to TO (°)	28.1 (6.3)	35.7 (7.2)	−7.6	**0.016**	**1.06**	32.3 (5.21)	36.1 (6.8)	−3.8	0.131	**0.56**
Moment Max (Nm/kg)	2.71 (0.49)	2.62 (0.55)	0.09	0.859	0.16	2.75 (0.38)	2.52 (0.58)	0.23	0.534	0.40
Moment change IC to MID (Nm/kg)	2.55 (0.53)	2.49 (0.53)	0.06	1.000	0.11	2.58 (0.37)	2.38 (0.60)	0.20	0.859	0.33
Moment change MID to TO (Nm/kg)	2.57 (0.58)	2.50 (0.55)	0.07	1.000	0.13	2.63 (0.45)	2.40 (0.63)	0.23	0.722	0.37
Knee										
Angle at IC (°)	13.5 (4.6)	18.7 (6.5)	−5.2	**0.026**	**0.80**	17.5 (4.5)	19.1 (7.0)	−1.6	0.929	0.23
Angle RoM IC to MID (°)	39.1 (4.7)	34.7 (3.4)	4.4	**0.033**	**1.29**	35.8 (4.1)	34.4 (4.6)	1.4	0.424	0.30
Angle RoM MID to TO (°)	26.5 (6.9)	26.4 (4.3)	0.1	0.534	0.02	26.8 (5.1)	28.3 (3.6)	−1.5	0.328	0.42
Moment Max (Nm/kg)	2.60 (0.64)	2.39 (0.65)	0.21	0.477	0.32	2.56 (0.47)	2.60 (0.60)	−0.04	0.859	0.07
Moment change IC to MID (Nm/kg)	2.92 (0.55)	2.64 (0.66)	0.28	0.155	0.42	3.03 (0.40)	2.84 (0.61)	0.19	0.374	0.31
Moment change MID to TO (Nm/kg)	2.35 (0.66)	2.17 (0.60)	0.18	0.424	0.30	2.49 (0.48)	2.45 (0.57)	0.04	0.594	0.07
Hip										
Angle at IC (°)	45.7 (7.2)	45.7 (6.7)	0.0	0.929	0.00	48.9 (7.4)	46.8 (7.1)	2.1	0.594	0.30
Angle RoM IC to MID (°)	3.4 (2.3)	6.6 (3.9)	−3.2	0.075	**0.82**	7.6 (4.7)	7.0 (4.1)	0.6	0.859	0.15
Angle RoM MID to TO (°)	34.0 (5.3)	32.0 (5.1)	2.0	0.374	0.39	33.9 (5.4)	34.1 (5.0)	−0.2	0.929	0.04
Moment Max (Nm/kg)	2.73 (0.59)	2.37 (0.85)	0.36	0.213	0.42	2.63 (0.45)	2.05 (0.72)	0.58	**0.050**	**0.81**
Moment change IC to MID (Nm/kg)	1.03 (0.72)	0.70 (0.60)	0.33	0.374	**0.55**	1.06 (0.61)	0.65 (0.70)	0.41	0.328	**0.59**
Moment change MID to TO (Nm/kg)	2.79 (0.78)	2.07 (0.75)	0.72	0.131	**0.96**	2.44 (0.63)	1.75 (0.74)	0.69	0.075	**0.93**
VGRF										
VGRF max (N)	1911 (290)	1819 (312)	92	0.328	0.29	1865 (259)	1820 (289)	45	0.534	0.16
Time to VGRF max (ms)	104 (11)	112 (6)	−8	**0.047**	**1.38**	112 (10)	116 (7)	−4	0.182	**0.58**

The bold values indicate statistical significance at the *p* = 0.05 level. In addition the bold values indicate - ES higher than 0.5 and represents a practically significant difference

There was a greater maximal net hip extension moment on the unaffected lower extremity of the AT group by 0.58 Nm/kg, 95% CI [−0.1, 1.3] compared to the matched lower extremity of the CTRL group (*P* ≤ 0.05; ES ≥ 0.5). Additionally, there was greater change in the net hip moment of the AT group for both lower extremities compared to the healthy control group during whole stance phase (Table 3, ES ≥ 0.5). There was a shorter stance time of the AT group by 8 ms, 95% CI [0.0, 20.0] compared to the control group (*P* ≤ 0.05; ES ≥ 0.5). Additionally, there was no maximal net ankle, knee or hip power difference between the groups (*P* ≥ 0.05; ES ≤ 0.5). The AT group exhibited greater asymmetry than the healthy control group for the ankle angle RoM from MID to TO, knee angle at IC, hip angle at IC, hip angle RoM from IC to MID and time to VGRF max (*P* ≤ 0.05) (see Table 3). Side to side asymmetries for all kinetic dependent measures exhibited no statistically significant differences between groups (*P* ≥ 0.05).

**Table 3:** Comparison of mean symmetry index (SI ± SD) during overground running between AT and healthy control group (Wilcoxon test, Effect of size)

Parameter	AT	Control	*Δ*	*P*	*ES*
Ankle					
Angle at IC	−0.43 (7.88)	0.84 (7.31)	−1.25	0.859	0.17
Angle RoM from IC to MID	−6.27 (75.45)	2.52 (27.60)	−8.79	0.657	0.32
Angle RoM from MID to TO	−15.17 (22.24)	−1.58 (10.64)	−13.59	**0.050**	**1.28**
Moment change from IC to MID	−2.43 (14.29)	5.56 (10.74)	−7.99	0.182	0.74
Moment change from MID to TO	−3.36 (14.93)	5.03 (10.78)	−8.39	0.182	0.78
Knee					
Angle at IC	−27.66 (27.14)	−2.24 (24.77)	−25.42	**0.008**	**1.03**
Angle RoM from IC to MID	8.87 (8.25)	1.24 (8.44)	7.63	0.110	**0.90**
Angle RoM from MID to TO	−2.68 (11.10)	−7.57 (10.33)	4.89	0.424	0.47
Moment change from IC to MID	−4.21 (11.86)	−8.22 (11.08)	4.01	0.374	0.36
Moment change from MID to TO	−8.07 (18.11)	−13.35 (12.34)	5.28	0.424	0.43
Hip					
Angle at IC	−6.78 (5.61)	−2.28 (5.63)	−4.50	**0.033**	**0.80**
Angle RoM from IC to MID	−77.55 (54.41)	−1.29 (31.92)	−76.26	**0.006**	**2.39**
Angle RoM from MID to TO	0.08 (19.55)	−6.82 (8.43)	6.90	0.248	**0.82**
Moment change from IC to MID	−15.72 (50.95)	17.16 (76.42)	−32.88	0.131	0.43
Moment change from MID to TO	12.71 (25.28)	18.08 (20.12)	−5.37	0.722	0.27
VGRF					
VGRF max	2.25 (2.36)	−0.33 (3.91)	2.58	0.110	0.66
Time to VGRF max	−7.85 (4.35)	−3.59 (4.24)	−4.26	**0.041**	**1.00**

The bold values indicate statistical significance at the *p* = 0.05 level. In addition the bold values indicate - ES higher than 0.5 and represents a practically significant difference

SI = ((Mean_affected/left_ − Mean_unaffected/right_)/0.5(Mean_affected/left_ + Mean_unaffected/right_)) ∗ 100

Angles – degrees; moments – Nm/kg, time – percent

## Discussion

The purpose of this study was to compare lower extremity mechanics of both the affected and unaffected limbs of AT ruptured runners with healthy controls. We found that participants with a previous AT rupture, compared to the healthy control group, did have: 1) a reduced ankle angle range of motion on the affected limb during push-off of the stance phase; 2) a reduced knee angle at initial contact and a greater knee range of motion.

Similar to previous reports [31], we found that participants from the AT group had an elongated Achilles tendon and an affected gastro-soleus complex and lower plantarflexion strength on affected side. We can speculate that an imbalance of the triceps surae tendon-muscular complex may influence the sagittal plane mechanics of the knee and ankle. To ensure sufficient tension of the elongated triceps surae complex during the initial ground contact, we can suggest two possible compensation mechanisms: 1) increased ankle dorsiflexion or 2) reduced knee flexion. Based on the results of this study, it appears that reduced knee flexion might be the preferred movement strategy used by the nine of the 11 AT individuals compared to the healthy controls (see Fig. 2). Although it was described in the case study of an athlete with a history of AT rupture [14], it appears that increased dorsiflexion is not preferred compensation mechanism in the AT individuals in this study. In the case of increased dorsiflexion, increased maximal vertical ground reaction forces during loading phase could occur. However, in this study there were no differences in the maximal vertical ground reaction force components of the AT group with reduced knee flexion.

Although compensation throughout reduced knee flexion might be advantageous to avoid a footfall pattern with a greater vertical ground reaction force component, this might not be a strategy without consequences [14]. Cooper and colleagues [32] suggested that knee hyperextension may provide a mechanism to control an unstable limb during the stance period of the gait cycle. However, hyperextension may place the knee at risk for injury of the capsular and ligamentous structures of the posterior aspect of the knee [32]. The gastrocnemius plays important role as stabilizer against overextension of the knee and anterior knee laxity [32, 33]. Overextension generally indicates the presence of an abnormal extension pattern following initial contact rather than the knee flexion pattern typical in healthy subjects [32]. The findings of the present study suggest that the AT repair results in an adaptation of the knee kinematics (i.e. reduced knee flexion). Whilst participants did not demonstrate hyperextension, there was less knee flexion during initial contact and thus a relatively extended knee that may predispose the runner to other injuries. An overextended knee might be a significant risk factor for an anterior cruciate ligament rupture particularly when landing from a jump or when transitioning from running to a cutting maneuver [34, 35]. However, this current research did not provide evidence that the participants with the AT repair demonstrated reduced knee flexion during landings or cutting maneuvers and this would need further evaluation.

The current study found that there was a 22% greater maximal hip joint moment on contralateral side of the AT group compared to the healthy controls. However, the 95% CI [−0.1, 1.3] suggests that difference 0.58 Nm/kg might not be significant. Increased joint moments on contralateral lower extremity may theoretically indicate increased load on contralateral side compared to the healthy controls. A study by Årøen et al. [12] found increased risk of contralateral AT rupture in AT ruptured population. In this study, Prilutsky et al. [28] demonstrated how proximal muscles transfer power to distal muscles via two joint muscles. Taking into account the two joint muscle transfer of power from hip to ankle via the gastrocnemius, we can speculate that an increased load on the AT of the contralateral side of the affected group would result [36]. However, with a non-significant increase in the ankle joint moments on contralateral side of AT group (0.23 Nm/kg; ES = 0.4), it seems that, instead of AT loading, there may be a strategy of increased contralateral hip loading during running of individuals with a history of AT rupture. This strategy of increased hip moments might be useful during low intensity activities. In this line, less ankle range of motion may actually lead to reduced affected limb ankle plantar flexion strength since this occurs during push off. A greater change in the net hip moment of the AT group compared to the healthy control group during the whole stance phase may be explained by reduced ankle plantar flexion strength on affected lower extremity.

The greater asymmetry of the AT group supports the results of the kinematics comparison between the AT affected and matched control limbs. The most interesting finding was that AT group exhibited higher asymmetry in the knee angle during initial contact. The present findings seem to be consistent with prospective studies that reported that previous injury is most the reported risk factor for running related injuries [37]. Nine of eleven AT individuals exhibited relative increased knee extension on injured side. In contrast, the healthy control group exhibited less asymmetry. It would seem that higher knee kinematic asymmetry of AT group may be related to a risk factor for injury of musculoskeletal system such as overextension.

This study has several imitations. First, the present study was designed as cross-sectional study and, as a result, we cannot determine if the findings of this study were the ‘cause’or the ‘result’ of the injury. A second limitation of this study concerns the Achilles tendon surgery performed on the individuals in the AT group. These individuals had their surgery performed by different surgeons who may have used different surgical techniques. A third limitation of this study concerns the length of time between the surgery and testing the participants. It is possible that the resulting altered running mechanics may have been an accommodation of the participants developing a reasonable strategy over the six years’ post-surgery to minimize the risk of injury.

## Conclusions

This study indicated that the individuals with history of AT rupture have reduced ankle range of motion during push-off phase of stance, reduced knee flexion during initial contact and an increased knee range of motion during the weight acceptance phase of stance on their affected limb compared to the healthy control group. These results suggest a compensation mechanism, overextending the knee at initial ground contact, against the deficit in the muscle-tendon complex of the triceps surae (i.e. elongation of AT). However, this compensation during sporting activities may place the knee at risk for further injury. Avoidance of AT lengthening and plantar-flexors structural and strength deficit after surgery or during the subsequent rehabilitation might help to manage AT rupture since that seems to be possible reasons for altered running kinematics. The results of this study indicate that individuals who have had an AT rupture should re-consider their participation in sports with high risk of AT injury and/or knee injury. In future studies, assessing the effect of AT rupture on the mechanics of the lower extremities during higher intensity movements than those presented in this study would be insightful.

## Abbreviations

AT: Achilles tendon
ATSR: Achilles tendon total rupture score
CTRL group: Control group
